# Internet addiction and associated factors among medical and allied health sciences students in northern Tanzania: a cross-sectional study

**DOI:** 10.1186/s40359-020-00439-9

**Published:** 2020-07-09

**Authors:** Innocent B. Mboya, Beatrice John Leyaro, Alberto Kongo, Charles Mkombe, Eliah Kyando, Johnston George

**Affiliations:** 1 Department of Epidemiology and Biostatistics, Institute of Public Health, Kilimanjaro Christian Medical University College, P. O. Box 2240, Moshi, Tanzania; 2 Department of Community Health, Institute of Public Health, Kilimanjaro Christian Medical University College, P. O. Box 2240, Moshi, Tanzania; 3 School of Mathematics, Statistics & Computer Science, University of KwaZulu Natal, Pietermaritzburg, Private Bag X01, Scottsville, 3209 South Africa

**Keywords:** Prevalence, Internet addiction, Internet addiction test, University students, Tanzania

## Abstract

**Background:**

Internet addiction is one of the fast-growing addictive behaviors and is a significant public health problem affecting a large number of people worldwide. Excessive use of the internet among university students increases their risk of internet addiction and related negative consequences. This study aimed to determine the prevalence and factors associated with internet addiction among medical and allied health sciences students in northern Tanzania.

**Methods:**

This cross-sectional study was conducted at Kilimanjaro Christian Medical University College (KCMUCo) from May to June 2018. A total of 500 consenting undergraduate students were sampled using the Simple Random Sampling technique proportional to the size of each class and a self-administered questionnaire used to collect data. Internet addiction was measured using a 20-item internet addiction test (IAT-20). Generalized linear model with Poisson family and log link function was used to estimate prevalence ratio (PR) and the corresponding 95% confidence intervals for factors associated with internet addiction.

**Results:**

The prevalence of internet addiction was 31%. Undergraduate students using the internet at the college were less likely to be addicted to internet (PR = 0.45, 95%CI 0.23, 0.86, *p* = 0.02) compared to those using both at the hostel/home and college. Higher prevalence of internet addiction was among those who used internet for a long time, i.e., 5 h or more per day (PR = 1.84, 95%CI 1.30, 2.63, *p* = 0.001) and for social networking (PR = 1.64, 95%CI 1.17, 2.31, *p* = 0.004) compared to those who used for less than 5 h per day and academic purposes, respectively.

**Conclusions:**

Thirty-one percent of undergraduate students in northern Tanzania were addicted to the internet. Internet addiction was associated with using the internet at the college, a long time using the internet per day, and social networking. The college administration should put effort into improving the accessibility, reliability, and regulation of college internet services to promote learning and academic productivity. We also recommend awareness creation intervention about the harmful consequences of excessive internet use in this population. More studies are needed to determine the burden of mental, psychological, and social problems associated with internet addiction in high-risk populations, and associated consequences.

## Background

Internet addiction is one of the fast-growing addictive behavior and is a significant public health problem, affecting a large number of people worldwide [1]. Repeated use of the internet leads to excessive use, usually termed as internet addiction or problematic internet use [2–6]. Internet addiction is a psychological dependence on the internet characterized by salience/excessive use, withdrawal, tolerance, negative repercussions/conflict, craving, and mood modification [1, 7, 8]. The use of the internet among students in universities and colleges has grown considerably [8–10]. Internet addiction among university/college students increases the risk of negative mental, physical, social, and financial consequences [1, 11, 12]. These include suicide, depression, psychological distress (depressive symptoms), anxiety, aggression, poor academic performance, cognitive impairment, poor dietary habits, lack of sleep, visual and hearing impairment, social isolation, and substance abuse such as cigarette smoking and alcohol use [2, 4, 11, 13–18]. The physical health consequences also include thromboembolism, musculoskeletal, ophthalmic, and metabolic problems [11]. These problems are likely to have devastating consequences to the wellbeing of students across universities in Tanzania, which may have a long-term impact on the social and economic welfare of the country.

The global burden of internet addiction among university students has not been established. However, studies in the USA have shown that 5–12% of students from different colleges have problematic internet use [4, 19]. In Asian countries, the prevalence ranged between 3.7% in India [20] to 37% in Malaysia and Iraq [9, 10] while in African countries, the prevalence ranged between 5.1% in Mauritius [21] to 16.8% in Kenya [22]. While the accessibility and use of the internet are high among university students in Tanzania [23], the burden of internet addiction is also not known.

Availability and use of the internet among university students are crucial in their academic endeavors [2]. However, other than academic activities, excessive gaming, internet-based gambling, sexual internet-based pre-occupations (such as viewing pornography), developing and sustaining intimate online relationships, entertainment and socializing or social networking through different social media platforms increase the odds of problematic internet use/ internet addiction [1, 2, 5, 9, 21]. Off-campus residence, longer time spent on the internet, frequency of internet use, and lower grade point average also have a significant influence on internet addiction in this population [4, 14, 16, 24]. There is conflicting evidence on the differences in internet addiction among male compared to female students [5, 17, 20–22, 25] though the former (males) are considered most at risk. In Tanzania, it is also unclear what factors increase the risk of internet addiction among university students, which have high access to internet services. The interplay of demographic, behavioral, social and academic factors may intensify the internet addiction problem and associated consequences in the student population.

Policies to limit accessibility, protect vulnerable individuals, internet addiction counseling, and addiction supervision centers have been established in some countries [1]. Tanzania has the national information and communications technologies policy in place that provides for legal and regulatory frameworks for the accessibility and use of the internet [26]. Current evidence is, therefore, crucial for informed policy decisions and for designing interventions to address problems associated with excessive use of the internet, especially among high-risk groups. This study aimed to determine the prevalence and factors associated with internet addiction among university students in northern Tanzania. On top of establishing the burden of internet addiction in this population, findings from this study may contribute to awareness creation on the associated risk factors and consequences, hence encourage students to take precautionary measures. Also, these findings may inform specific interventions by respective or other Universities/ colleges to promote better learning experiences among their students and inform the public health interventions to improve mental health and wellbeing in the country.

## Methods

### Study design and setting

We carried out a cross-sectional study between May and June 2018 at Kilimanjaro Christian Medical University College (KCMUCo) located in Moshi Municipality, Kilimanjaro region in North-eastern Tanzania. KCMUCo is among the four universities in Moshi Municipality, northern Tanzania, and is the only university in the region offering medical education and other allied health programs. Programs offered in this higher learning institution include Diploma and Bachelor’s in health laboratories, Bachelor in Nursing, Diploma in Occupation Therapy, Diploma and Bachelor in Physiotherapy, Diploma and Bachelor in Prosthetics and Orthotics, Doctor of Medicine, Bachelor in Optometry and Diploma in HIV and AIDS care. The college had about 1600 undergraduate students between the year 2018–2019.

### Study population, sample size, and sampling technique

The study population and inclusion criteria were the registered undergraduate medical and allied health sciences students at KCMUCo who were in class during the data collection period and provided their informed consent. We used a single proportion formula for sample size calculation using a standard normal value of 1.96 at 95% confidence interval, 3.5% margin of error, and 16.8% prevalence of internet addiction among university students in Kenya [22]. After adding 10% of non-response, a minimum required sample size was 500 students. We applied a simple random sampling technique using probability proportional to the size of each class to sample students from their respective classes.

### Study variables

The dependent variable in this study was internet addiction, assessed using the Internet Addiction Test (IAT-20) score. This tool is a 20-item 5-point Likert scale self-reporting tool used to assess internet addiction and its severity. This tool has been validated and used in other parts of the world [7, 14, 19, 27], but has not been validated in Tanzania. We calculated the IAT score by adding the scores obtained for all 20 items, with a total ranging from 0 to 100 [17]. After computing scores for each question, we categorized internet addiction into a binary variable whereby those with a score of 50 and above were considered addicted to the internet and not addicted if otherwise. The independent variables included socio-demographical characteristics, particularly age in years, year of study, sex, marital status, and area of residence. Internet use practices included gadgets for internet use (college computers, personal/ own device or a combination of these two), method of internet access (College Wi-Fi, mobile data/ personal hotspot, or both), amount of money in Tanzania shillings spent on mobile data in a typical day, a regular place for internet use (hostel/ home, college, or both), time in hours spent using internet per day, and primary purpose using the internet (academic/ social networking and surfing).

### Data collection methods, tools, and procedures

A self-administered questionnaire was used for data collection. The questionnaire collected information on participant social-demographic characteristics, internet use practices, the purpose of internet use, and the Internet Addiction Test. The questionnaire was in English, an official language at higher learning institutions in Tanzania. Trained medical students at KCMUCo (third to fifth authors) collected data. Data collectors visited students in their respective classes and clearly explained the purpose of the study. After addressing all questions related to this study, students were handled over the questionnaire and asked to respond in about 15–20 min. Data collectors were available to ensure that participants respond to questions themselves (given that they were in class with fellow students) and also clarified any raised concerns. Finally, data collectors thanked respondents for their time and cooperation. All completed questionnaires were then collected and revised for completeness.

### Statistical analysis

We performed data cleaning and analysis using Stata version 15.1. Categorical variables were summarized using frequency and percentages, while the median and interquartile range used to summarize numeric variables. Using Cronbach’s Alpha statistic, we assessed the reliability of the IAT-20 items. Generalized linear model with Poisson family and log link function was used to estimate prevalence ratio (PR) and the corresponding 95% confidence intervals for factors associated with internet addiction. We developed several adjusted analyses models to determine factors independently associated with internet addiction using stepwise regression. The pairwise correlation assessed the collinearity between variables included in the analysis. We used the likelihood ratio test to evaluate the effect of either retaining or removing age and sex in the multivariable analysis being the known confounders. A mode without these variables was considered the best fit.

## Results

### Characteristics of study participants

The mean age of 500 respondents was 23.8, with a standard deviation of 2.4 years, and about three quarters were aged ≤24 years. More than half (58.4%) of all respondents were males, and 60% were from the doctor of medicine program. Only 6.2% were in union/ married, 89.2% resided off-campus, and over 92.2% were beneficiaries of the Higher Education Student Loans Board (HESLB), with only three receiving private sponsorship (Table 1).

**Table 1:** Characteristics of study participants *(N = 500)*

Variables	Frequency	Percentage
**Age in years**		
Mean (SD)	23.8 (2.4)	
≤ 24	365	73.0
> 24	135	27.0
**Sex**		
Male	292	58.4
Female	208	41.6
**Program of study**		
Doctor of medicine	298	59.6
Nursing	90	18.0
Laboratory science	51	10.2
Physiotherapy	35	7.0
Optometry	12	2.4
Prosthetics and orthotics	14	2.8
**Year of study**		
Year 2	144	28.0
Year 3	216	43.0
Year 4	87	17.4
Year 5	53	10.6
**Marital status**		
In union	31	6.2
Not in union	469	93.8
**Residence**		
On-campus	54	10.8
Off-campus	446	89.2
**Source of fund**		
Family/personal	39	7.8
HESLB/Sponsor	461	92.2

### Internet use practices

Seventy percent of all respondents access the internet using personal devices, i.e., mobile phones, laptops, and tablets. Half (51%) access the internet using both own devices and college computers. The median amount of money spent on mobile data in a typical day was 500 Tanzanian shillings, equivalent to 0.22USD [given 1 USD = 2300 Tanzanian Shillings], and more than half (56.4%) spent ≤500 Tshs per day. Sixty percent of all respondents regularly use the internet both at hostels/home and the college. The median time (IQR) spent on the internet on a typical day was 5 (3, 8) hours, and 44.8% spent < 5 h a day. Academic reasons (56.4%) were the primary purpose of internet use in this population (Table 2).

**Table 2:** Internet use practices *(N = 500)*

Internet use practices	Frequency	Percentage
**Gadget for internet use**		
College computers	8	1.6
Personal/Own device	351	70.2
Mixed	141	28.2
**Method of internet access**		
College Wi-Fi alone	15	3.0
Mobile data/personal hotspot	230	46.0
All the above	255	51.0
**Amount (Tshs) spent using mobile data in a day**		
Median (IQR)	500 (500, 1000)	
≤ 500	282	56.4
> 500	218	43.6
**Commonplace of internet use**		
Hostel/home	133	26.6
College	70	14.0
Both hostel/home and college	297	59.4
**Time (hours) spent using the internet per day**		
Median (IQR)	5 (3, 8)	
< 5	224	44.8
≥ 5	276	55.2
**The primary purpose of internet use**		
Academic	282	56.4
Social networking	155	31.0
Internet surfing	63	12.6

### Prevalence of internet addiction

The median internet addiction score was 38 and interquartile range of 26–53. The Cronbach’s alpha test for the reliability of the 20 items used to measure internet addition provided a scale of 91.1%, indicating the test is reliable. The overall prevalence of internet addiction among 500 undergraduate students was 31%.

### Unadjusted analysis for factors associated with internet addiction

Factors associated with internet addiction in the unadjusted analysis were the place for internet use, time spent using the internet per day, and primary purpose for internet use (Table 3). The prevalence of internet addiction was 14.3% among students using the internet through college premises, 40.2% among those spending ≥5 h on the internet per day, and 44.5% among those who used the internet for social networking. Prevalence was significantly lower (PR = 0.40, 95%CI 0.21, 0.77, *p* = 0.01) among those using the internet at the college compared to those using both at the hostel/home and college. In contrast, those who used the internet for a long time, i.e., 5 h or more per day were more likely to have internet addiction (PR = 2.05, 95%CI 1.44, 2.90, *p* < 0.001) compared to those used for less than 5 h per day. Also, a higher prevalence of internet addiction was among those using the internet for social networking (PR = 1.87, 95%CI 1.34, 2.62, *p* < 0.001) compared to those used for academic purposes.

**Table 3:** Unadjusted analysis for factors associated with internet addiction *(N = 500)*

Variables	n	Addicted Yes (%)	CPR^**a**^ (95%CI)	***P-value***
**Age (years)**				
≤ 24	365	116 (31.8)	1.10 (0.77, 1.58)	0.61
> 24	135	39 (28.9)	1.00	
**Sex**				
Male	292	100 (34.2)	1.00	
Female	208	55 (26.4)	0.77 (0.56, 1.07)	0.12
**Year of study**				
Year 2	144	41 (28.5)	1.00	
Year 3	216	75 (34.7)	1.22 (0.83, 1.78)	0.31
Year 4	87	20 (23.0)	0.81 (0.47, 1.38)	0.43
Year 5	53	19 (35.8)	1.26 (0.73, 2.17)	0.41
**Marital status**				
In union	31	7 (22.6)	0.72 (0.34, 1.53)	0.39
Not in union	469	148 (31.6)	1.00	
**Residence**				
On-campus	54	18 (33.3)	1.00	
Off-campus	446	137 (30.7)	0.92 (0.56, 1.51)	0.74
**Source of fund**				
Family/personal	39	9 (23.1)	1.00	
HESLB/Sponsor	461	146 (31.7)	1.37 (0.70, 2.69)	0.36
**Gadget for internet use**				
College computers	8	1 (12.5)	0.43 (0.06, 3.13)	0.40
Personal device	351	113 (32.2)	1.11 (0.77, 1.58)	0.58
Mixed	141	41 (29.1)	1.00	
**Method of internet access**				
College Wi-Fi alone	16	2 (12.5)	0.43 (0.11, 1.74)	0.24
Mobile data/personal hotspot	230	79 (34.3)	1.18 (0.86, 1.62)	0.31
All the above	254	74 (29.1)	1.00	
**Commonplace of internet use**				
Hostel/home	133	39 (29.3)	0.82 (0.57, 1.19)	0.29
College	70	10 (14.3)	0.40 (0.21, 0.77)	0.01
Both hostel/home and college	297	106 (35.7)	1.00	
**Amount (Tshs) spent using mobile data in a day**				
≤ 500	282	85 (30.1)	1.00	
> 500	218	70 (32.1)	1.07 (0.78, 1.46)	0.70
**Time (hours) spent using the internet per day**				
< 5	224	44 (19.6)	1.00	
≥ 5	276	111 (40.2)	2.05 (1.44, 2.90)	< 0.001
**Primary purpose for internet use**				
Academic	282	67 (23.8)		
Social networking	155	69 (44.5)	1.87 (1.34, 2.62)	< 0.001
Internet surfing	63	19 (30.2)	1.27 (0.76, 2.11)	0.36

^a^ Crude prevalence ratio

### Adjusted analysis for factors associated with internet addiction

In multivariable analysis, factors that remained to be significantly (*p* < 0.05) associated with internet addiction were commonplace for internet use, average time spent using the internet per day and primary purpose for internet use. Undergraduate students using the internet at the college were less likely to have internet addiction (PR = 0.45, 95%CI 0.23, 0.86, *p* = 0.02) compared to those who used both at the hostel/home and college. Higher prevalence of internet addiction was among students who used internet for a long time, i.e., 5 h or more per day (PR = 1.84, 95%CI 1.30, 2.63, *p* = 0.001) and for social networking (PR = 1.64, 95%CI 1.17, 2.31, *p* = 0.004) compared to those used for less than 5 h per day and for academic purposes (Table 4).

**Table 4:** Adjusted analysis for factors associated with internet addiction (*N* = 500)

Variable	APR^**a**^	95%CI	***P-value***
**Regular place for internet use**			
Hostel/home	0.82	(0.57, 1.18)	0.28
College	0.45	(0.23, 0.86)	0.02
Both hostel/home and college	1.00		
**Time (hours) spent using the internet per day**			
< 5	1.00		
≥ 5	1.84	(1.30, 2.63)	0.001
**Primary purpose for internet use**			
Academic	1.00		
Social networking	1.64	(1.17, 2.31)	0.004
Internet surfing	1.15	(0.69, 1.92)	0.59

^a^ Adjusted prevalence ratio, adjusted for a regular place for internet use, average time spent using the internet per day and primary purpose for internet use

## Discussion

In this study, the prevalence of internet addiction was 31%. Internet addiction was significantly associated with using internet at the college, spending 5 h or more using the internet per day, and using the internet for social networking. The prevalence of internet addiction in this study was higher than that reported from the USA [4, 19], Asia [20], and among African countries [21, 22]. A slightly higher prevalence (37%) was reported in Malaysia and Iraq [9, 10] as well as (40.7%) in Iran [27]. Variations in prevalence could be due to differences in the tools used. For instance, a study in Iraq [10] utilized an 8-item scale as opposed to the 20-item scale used in this study. In contrast, [16, 21] utilized a modified version of the scale, and [5] used a different tool (i.e., Chen Internet Addiction Scale) to assess internet addiction. Higher prevalence may also be attributed to internet access using personal devices, considering that more than 70% of all respondents in this study accessed the internet using their mobile devices.

The higher prevalence of IA in this study may have serious implications for both social, mental, and physical health as well as financial consequences. The combined effect of these problems could have serious implications for academic performance [2, 16, 28, 29]. For example, problems with self-care, difficulty in performing daily routine, pain and discomfort, anxiety, and depression were the consequences of internet addiction reported among young Vietnamese population (15–25 years of age) comprising a mixture of high school and university students [18]. Other literature reported consistent findings, which also included additional critical mental, physical, and social problems associated with internet addiction among university students [1, 3, 4, 9, 11, 15, 17, 22, 24]. Internet addiction problems should, therefore, be addressed both as a public health and clinical problem focusing on reducing potential negative psychosocial consequences of risky/ harmful content, excessive/ maladaptive use, and financial burden [11], particularly on the high-risk populations. Mental health research in Tanzania is limited. We recommend more studies to assess the potential risk factors, consequences, and cost-effective as well as context-specific interventions to address the common mental health problems in the country, such as the alarming prevalence of internet addiction.

In this study, university students spending a long time (more than 5 h per day) on the internet increased the likelihood of internet addiction, which is consistent with findings from other studies [14, 16, 21, 24]. An excessive amount of time spent on the internet serves as a distraction from schoolwork. This situation puts students at a disadvantage in as far as academic productivity is involved [16]. If used positively, the internet is potentially useful and informative both academically and socio-economically, and it is capable of assisting university students to achieve their lifetime goals [2, 22].

University students who reported using the internet for social networking were more likely to be addicted to the internet compared to those who used it for academic purposes. This finding concurs with other studies [2, 9, 16, 17, 21, 22, 30]. Like in other studies, the most commonly used social media platforms in this population included Instagram, YouTube, Snapchat, Skype, WhatsApp, and Facebook, among others [2, 30, 31]. These students could, therefore, be spending less time online engaging in academic activities, which are likely to affect their academic productivity or performance [2, 16, 28], which increases the risk of mental, psychosocial and physical consequences.

University students who were using the internet at the college were less likely to have internet addiction compared to those using the internet both at home/ hostel and college. In India, university students who stayed in rented accommodations were more likely to be addicted to the internet [14]. Internet use through the college facilities might offer some protection against excessive use, by restricting the contents to academic-related materials. Concerted efforts by the college should ensure the availability, reliability, and regulation of college internet services to encourage learning and enhance academic performance, particularly on the campus premises.

While the current study has provided an overview of the current status of the burden of internet addiction among university students in Tanzania, it has several limitations. Firstly, this study used a cross-sectional design that does not allow drawing any inference on the cause-effect relationship of internet addiction and associated factors. Secondly, the study might have missed students who were severely addicted to the internet as they are more likely to miss classes. The tool utilized for data collection is a self-reported measure to assess internet addiction; hence is subject to potential information bias, particularly recall and self-desirability bias. Also, this tool has not been validated in the Tanzanian context, highlighting a need for a validation study. Lastly, these findings may not be generalized to students in other universities or the general population across the country.

## Conclusions

Thirty-one percent of medical and allied health sciences students in northern Tanzania were addicted to the internet. Internet addiction was associated with using the internet at the college, spending more time using the internet per day, and using the internet for social networking. The college administration should put effort into improving the accessibility, reliability, and regulation of college internet services that may promote learning, and academic productivity. We also recommend awareness creation intervention about the harmful consequences of excessive internet use in this population. More studies are needed to determine the burden of mental, psychological, and social problems associated with internet addiction in high-risk populations, and associated consequences. Furthermore, to inform policy decisions and interventions, there is a need to intensify research studies on mental health issues in Tanzania.

## Data Availability

The datasets generated and/ or analyzed during the current study are available in the Mendeley Data repository, 10.17632/8thfmxwmm4.1.

## Abbreviations

IAT: Internet Addiction Test
KCMUCo: Kilimanjaro Christian Medical University College
PR: Prevalence ratio
